# Ethics in the interface between multidisciplinary teams: a narrative in stages for inter-professional education

**DOI:** 10.1080/17571472.2016.1244892

**Published:** 2016-10-24

**Authors:** Katherine Wiles, Nawal Bahal, Hilary Engward, Andrew Papanikitas

**Affiliations:** ^a^ McKinsey and Company, London, UK; ^b^ Anaesthetics and Acute Pain, Buckinghamshire Healthcare NHS Trust, Aylesbury, UK; ^c^ Medical and Healthcare Education, Veterans and Families Institute, Anglia Health Partnership, Faculty Medical Science, Postgraduate Medical Institute, Anglia Ruskin University, Chelmsford, UK; ^d^ NIHR Academic Clinical Lecturer, Nuffield Department of Primary Care Health Sciences, New Radcliffe House, Radcliffe Observatory Quarter, University of Oxford, Oxford, UK

**Keywords:** Ethics, education, narrative, inter-professional, multi-disciplinary

## Abstract

An ethically problematic clinical case is used to illustrate the potential importance of understanding clinical ethics in an interdisciplinary context. Whilst much has been written on ethics education for multidisciplinary and interdisciplinary teams, we argue that it is important that both healthcare professions and healthcare teams are able to look outside their own disciplinary ethos and sometimes outside their formal teams when considering the ramifications of an ethical issue. A complex (fictional but based on the authors’ pooled experiences) case involving the delivery of a new-born from a mother with HIV is used to illustrate this, because multiple clinical teams will be involved at different times and in parallel with one another.

## Why this matters to me

All the authors work with multidisciplinary and interdisciplinary teams. We believe that better understanding of the inter-professional aspects of ethical decision-making may lead to both better teamwork and potentially improved patient outcomes and morale in healthcare workers.

## Key messages

• Moral leadership in healthcare can be expressed by any member of the healthcare team, however junior.
• Awareness of national/international context, professional guidelines and legislation can be useful in cross-boundary discussions.
• Better understanding of the inter-professional aspects of ethical decision-making may lead to both better teamwork and potentially improved patient outcomes and morale in healthcare workers.

## Introduction

Ethics has been a recent feature in inter-professional education for healthcare undergraduates on the basis that even if knowledge and technical skills are not shared, ethics and values can be. This approach at worst can be used to justify ‘dry’ philosophy lectures to a diverse healthcare audience who all find it equally irrelevant or a simplistic and superficial approach to ethics which makes it easy for diverse groups to sign up to a common but superficially understood set of values. Thankfully the approaches currently being taken to ethics in inter-professional education are highly nuanced, encourage critical but respectful inter-professional engagement and include input into postgraduate healthcare education and continuing professional development.[1]

In this paper we offer a clinical narrative as an illustration of how ethical issues affect many members of the wider healthcare team. We suggest that such a case may be used as a tool for teaching students from a variety of healthcare disciplines together. We aim to illustrate how mutual understanding of the different team members’ perspectives might lead to a more thoughtful team approach.

We have constructed a teaching case that involves several professions and also more than one kind of team. This is relatively innovative – in their seminal paper on inter-professional ethics, Clark et al. suggest that published discussion about inter-professional ethics is highlighted only in particular teams and is often restricted to surgical teams, intensive care teams, and mental health teams. We suggest that there are other team scenarios where inter-professional ethics may be discussed, and that close knit interdisciplinary teams working to a very clear purpose may offer a different experience to the more disparate teams seen in primary healthcare.[2] There is clear importance in recognising the different values-perspectives of the differing professions on the team.[3–6] Clark et al’s widely cited approach provides a framework for analysis of the inter-professional tensions and ethical nuances by discussing factors that affect ethical decision-making on two axes.[4] On one axis they discuss: ethical principles (e.g. the four ethical principles of Beauchamp and Childress), structures (Structures are established patterns of thought and behaviour within an organisation for individual and collective practices related to teamwork) and processes (the actual content or activities that occur within the structures previously discussed). On the other axis they discuss: the individual healthcare worker, the inter-professional team, and the organisation.

In Clark et al’s paper, the ‘principles’ refers to qualitative differences in the moral commitments and priorities of profession, in tension within a team, as might be manifest in doctors preferring a good balance of benefit over harm, nurses valuing caring and ministering to distress, and social workers prioritising social justice. Good empirical studies showcasing this phenomenon are lacking and we consider this to be at best a caricature, but it makes a reasonable educational point nonetheless. ‘Structures’ refers to thinking about differences in education and enculturation of healthcare workers – such as different interest, university versus non-university based training etc. This can shape the nature of the discourse, as highlighted by Holm’s analysis of ethics talk in a mixed inter-professional group – doctors did most of the talking and nurses did most of the consensus-building.[7] Processes are equally critical in the shaping of each professional’s moral agency. This is highlighted by Wintrup’s case of doctors prescribing oral fluids on a drug chart so that nurses who have no time to help ward patients drink are forced to make it a priority.[8] The external factors which enhance or inhibit teamwork and individual agency are relevant here.

**Figure 1.**
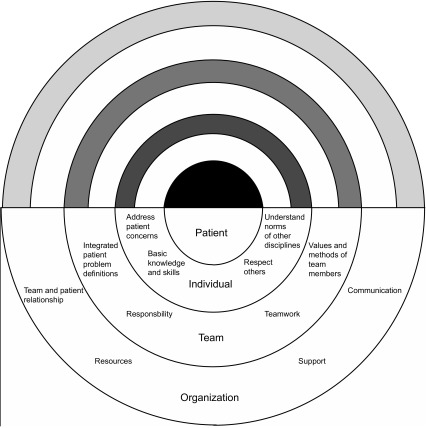
The roles of individuals, teams, and organisations in the care of a patient.

Our case is presented in the context of the UK healthcare system and so relies upon UK law and ethical norms. Such a system may not apply internationally. However, the authors contend that principles of how the inter-professional team can navigate ethical dilemmas have international relevance (Figure 1).

## Inter-professional ethics case

*We suggest that this case is reflected on in stages.*

**Stage 1:** An on call anaesthetics registrar questions the indication for an elective caesarean section to be performed out of hours, with universal precautions such as double-gloving of all clinical staff and the wearing of goggles. The consultant obstetrician informs him that the mother is HIV positive. However he is told that under no circumstances must this information be shared with any but the core clinical staff involved as the husband is unaware of her infectious status. The patient has stated that she contracted the virus as a consequence of rape subsequent to becoming pregnant. The midwife explains that the patient and her husband are from sub-Saharan Africa. The patient is concerned that disclosure may result in violence against her and abandonment of herself and the baby by the father and other family members. Furthermore the stigma associated with rape may in itself prompt the husband to abandon her. Moreover, she *is expected* to breastfeed. In the region in which she was raised, only the women with HIV/AIDS did not breastfeed. The genito-urinary medicine (GUM) team have been monitoring the situation, and there is a special code in the maternal handheld notes to alert treating clinicians of her infection. Thus far the mother has been able to conceal her antiretroviral therapy and GUM clinic attendances from her husband.

**Stage 2:** It also transpires that her diagnosis took place at a GUM clinic. Her GP and dentist do not know of her HIV positive status. She states that this is because she lives in a small village and that confidentiality is ‘impossible’. The anaesthetics speciality registrar and obstetrics trainee have a sense of unease over the case. Something does not ‘add up’. The elective Caesarean section is uneventful, and a vigorous boy is born who cries immediately. A nursing student who is party to the team discussion volunteers that she remembers the patient from a casualty attendance 2 years previously when she was not pregnant. She states that she remembers that they had to respect the patient’s confidentiality over HIV at that time. The anaesthetist looks through the hospital notes, and discovers that the patient’s HIV status precedes her pregnancy. She has maintained throughout that the child is her husband’s. Even if it were not, it is unlikely that she has not had sexual relations with her husband, as he appears certain he has fathered the child.

**Stage 3:** The anaesthetist does not feel it is his job to follow-up the perceived ethical dilemma as his intervention, a spinal anaesthetic, is complete and the mother is now on the postnatal ward. The on-call consultant obstetrician is reluctant to pursue matters, as he feels that the GUM doctors have ‘ownership’ of the problem. The gynaecology trainee directed to a communicable diseases nurse practitioner who is the liaison for the GUM team who has assured him that the mother has been told twice that she has an obligation to tell her partner, but that there the obligations of the healthcare professions end. He has also spoken to the trust lawyer, who has said this is a clinical matter and not a legal one.

**Stage 4:** In the meantime, the mother declines an HIV test for her newborn and states that she would like to breastfeed. At this point the obstetrics trainee, still troubled, has contacted a medical defence organisation. They recommend disclosure. He decides to talk to the head of women’s and children’s services at the hospital, a consultant paediatrician. The paediatrician tells the mother that the hospital doctors have a duty to her, but they also now have a duty to her husband and son. He arranges a case conference with the mother, senior midwife, and the nurse practitioner of the GUM team. The purpose is to support the mother telling her husband. She is informed however that if she does not tell her husband a member of the women’s and children’s services team will be obliged to do so.

**Stage 5:** The conference with the father takes place. In the end the paediatrician has to tell the husband. He tries to assault the mother and security guards are called. He later leaves her and the child, but takes an HIV test. When this is positive, he disappears from follow up, but not before he gives the names of four other women he was sleeping with after his wife became pregnant. The child’s test comes back positive …

Or …

The conference takes place. The patient tells her husband. He is surprised and sad, but says he will stand by her. They come up with a more coherent strategy to let key health workers, such as her dentist, know of her status. The child’s HIV test comes back negative.

## Discussion

Various authors use cases to illustrate inter-professional ethics in different ways. Clark et al. use a reasonably detailed case but focus on inter-professional tensions in a team’s way of working rather than on dilemmas in a particular patient’s care.[4] By contrast King offers two contrasting examples of inter-professional teamwork working well and badly in the primary healthcare setting.[9] In King’s cases the teams that work well are adequately resourced and have ways of communicating with one another. Spike and Lunstroth offer a set of brief cases based on their overlapping relevance to particular professions.[10] We have opted to share a more detailed case in the hope that this will both offer an element of authenticity and generate more avenues for discussion. We suggest that each stage can be used to start discussion about and revisit core elements of interdisciplinary clinical ethics. Nothing about any of the character’s moral positions should be taken for granted – just because a profession has focus on caring does not mean that a particular member of that profession cares. The focus ought to be the patient but it there should also be some focus on the team as well.[2] A class may use a framework to identify issues such as the four principles, Jonsen’s ethical grid [11] or a more complex grid such as that of Seedhouse.[12] We suggest that having multiple endings is a good way to illustrate the idea that members of a team can act in a robustly ethical manner with disastrous results, or be neglectful in their ethical duties without significant harm arising in a specific case. This means that rightness and wrongness are not necessarily determined by the actual outcome but by the right decisions behaviours displayed by the team in a given case such as the one above.

Applying a framework such as that used by Clark et al. enables us to see that each team involved might have different primary duties and concerns. For example, the anaesthetist might be primary concerned with the safety of administering the anaesthetic and the safety of the staff involved in the operation. By contrast the genitourinary medicine team may be primarily concerned with the mother’s health and protecting confidentiality so that people with sexually transmitted diseases are not deterred from accessing their service. Acting outside of these duties involves some effort – the actions of the junior clinicians in the above case arguably overcome some of the limitations of structures and processes. Moreover they make use of other social structures which exist to clarify professional duty and patient safety. Examples of this in the above case include the informal collegiality of junior doctors and the relative formality of a multidisciplinary meeting. While clinicians are certainly a large part of discussions surrounding patient care, there is potential for individual members of an inter-disciplinary team to stay focused on their specific tasks and their contribution may be in fact only documenting how their individual skills were employed. This can create potential gaps in care, illustrated in recent failures to address child abuse in the UK.[13]

## Conclusion

Respect (in a spirit of critical friendship) for different values and ethical positions held by various team members (which may or may not be negotiable) are what contribute to a successful team. A team that can air disagreements has a fighting chance of resolving them.[14] A team with diversity in their moral gaze, we suggest, will have members that can see relevant moral issues that their colleagues may miss. Conflict, however, may still arise even if all members feel that they are working towards the same goal. This is because goals and values are variously interpreted in ways that are individual, professional and cultural. For example, contextual features [11] may include both the institutional ethos and ethical priorities of each individual involved. Importantly this may mean that there are different perspectives in tension even within a team that is uni-disciplinary. In any case we argue that there is benefit in having a conceptual tool that enables us to recognise, and also learn from, the differences in how different professions might talk about and enact ethics. It may also crucially involve a discussion of the agency of each team member: what ability does each actor in this narrative have to make a decision or influence the outcome? The final stage might be a discussion of whether the outcome determines the rightness or wrongness of the clinical decisions.[15] We welcome use of this case in further discussion and in teaching.

## Governance

This paper uses a fictional case developed from the authors’ pooled experience and expertise. The authors take personal responsibility for the work.

## Funding

AP is funded by the National Institute of Healthcare Research to research postgraduate and primary care ethics education.

## Disclosure statement

No potential conflict of interest was reported by the authors.

## Acknowledgements

We would like to thank the following people for their comments on the manuscript: Professor Paul Thomas, Dr. John Spicer and Dr. Peter Toon

## References

[CIT0001] McAuliffe D (2014). Interprofessional ethics: collaboration in the social, health and human services.

[CIT0002] Engward H, Papanikitas A, Spicer J Interprofessional ethics in the primary care setting. Handbook of primary care ethics.

[CIT0003] Aveyard H, Edwards S, West S (2005). Core topics of health care ethics. The identification of core topics for interprofessional education. J Interprof Care.

[CIT0004] Clark PG, Cott C, Drinka TJ (2007). Theory and practice in interprofessional ethics: a framework for understanding ethical issues in health care teams. J Interprof Care.

[CIT0005] Hall P (2005). Interprofessional teamwork: professional cultures as barriers. J Interprof Care.

[CIT0006] Wachter M (1976). Interdisciplinary teamwork. J Med Ethics.

[CIT0007] Holm S, Gjersoe P, Grode G Ethical reasoning in mixed nurse-physician groups. J Med Ethics.

[CIT0008] Wintrup J The changing landscape of care: does ethics education have a new role to play in health practice?. BMC Med Ethics.

[CIT0009] King A, Bowman D, Spicer J (2007). Interprofessional teamworking: a moral endeavour? An exploration of clinical practice using Seedhouse’s ethical grid. Primary care Ethics.

[CIT0010] Spike J, Lunstroth R (2016). A casebook in interprofessional ethics: a succinct introduction to ethics for the health professions.

[CIT0011] Jonsen AR, Siegler M, Winslade WJ Clinical ethics: a practical approach to ethical decisions in clinical medicine1982.

[CIT0012] Seedhouse D (2002). Commitment to health: a shared ethical bond between professions. J Interprof Care.

[CIT0013] Hall D Child protection–lessons from Victoria Climbie. Bmj.

[CIT0014] Tuckman BW (1965). Developmental sequence in small groups. Psychol Bull.

[CIT0015] Gillon R (2003). Ethics needs principles–four can encompass the rest–and respect for autonomy should be “first among equals”. J Med Ethics.

